# Reflectance confocal microscopy - Consensus terminology glossary in Brazilian Portuguese for normal skin, melanocytic and non-melanocytic lesions^☆^

**DOI:** 10.1016/j.abd.2023.05.001

**Published:** 2023-09-28

**Authors:** Juliana Casagrande Tavoloni Braga, Carlos B. Barcaui, Ana Maria Pinheiro, Ana Maria Fagundes Sortino, Cristina Martinez Zugaib Abdalla, Gabriella Campos-do-Carmo, Gisele Gargantini Rezze, Juan Piñeiro-Maceira, Lilian Licarião Rocha, Marcus Maia, Bianca Costa Soares de Sá

**Affiliations:** a Department of Dermatology, Núcleo de Câncer de Pele, A.C. Camargo Câncer Center, São Paulo, SP, Brazil; b Service of Dermatology, Universidade do Estado do Rio de Janeiro, Rio de Janeiro, RJ, Brazil; c Department of Dermatology, Universidade de Brasília, Brasília, DF, Brazil; d Department of Dermatology, Hospital Sírio Libanês, São Paulo, SP, Brazil; e Department of Dermatology, Instituto Nacional de Câncer, Gávea Medical Center, Rio de Janeiro, RJ, Brazil; f Department of Dermatology, Hospital Clínic I Provincial da Universidade de Barcelona, Barcelona, Spain; g Department of Dermatology, Hospital das Clínicas, Faculty of Medicine, Universidade de São Paulo, São Paulo, SP, Brazil; h Dermatology Clinic, Santa Casa de Misericórdia São Paulo, São Paulo, SP, Brazil

**Keywords:** Adnexal and skin appendage, Diagnostic imaging, Melanoma, Microscopy, confocal, Neoplasms, Skin, Terminology

## Abstract

**Background:**

Currently, there is no uniform and official terminology in Portuguese for reflectance confocal microscopy analysis, despite the increasing number of Brazilian dermatologists using this new tool.

**Objective:**

To present the terminology in Brazilian Portuguese for the description of reflectance confocal microscopy and establish the first Brazilian consensus on terms related to normal skin and cutaneous tumors.

**Methods:**

10 Brazilian specialists from different institutions and states of Brazil were selected to evaluate the best corresponding terms in Portuguese for normal skin, melanocytic and non-melanocytic tumors. The terms used were translated from international consensuses in the English language. The modified Delphi method was used to create the consensus in 3 steps.

**Results:**

The terms considered the most appropriate in the Portuguese language to describe the findings of normal skin, melanocytic and non-melanocytic lesions in the reflectance confocal microscopy analysis were presented.

**Study limitations:**

The limitations of the present study include the number of participants and limited regional representation (only two of the five Brazilian regions were represented).

**Conclusion:**

This Brazilian consensus represents an opportunity for dermatologists and physicians specializing in cutaneous oncology to become familiar with reflectance confocal microscopy, propagating the technique in clinical and research environments to stimulate national and international publications on this subject.

## Introduction

Several technologies are currently available to aid in the evaluation and diagnosis of skin lesions, such as ultrasonography, *in vivo* reflectance confocal microscopy (RCM), *ex vivo* confocal microscopy, magnetic resonance imaging, spectroscopy, and optical coherence tomography, among others.

RCM is a recently introduced non-invasive imaging method that allows real-time skin examination with morphological assessment with *in vivo* cellular and nuclear resolution, without the need for fluorescent markers or tissue dyes.^1^

Image depth is limited to about 200 µm, which usually allows examination down to the papillary dermis. This depth routinely includes and allows examination of the dermal-epidermal junction (DEJ), which is generally located at 50 to 150 µm depth. For dermatologists and pathologists, the DEJ is of great interest, as most skin cancers originate in the basal layer and DEJ.^2^ However, this depth limit may hinder the observation of structures of interest for the evaluation of tumors in special areas such as the palmo-plantar surface, thick, hyperkeratotic, and ulcerated lesions.

The imaging analysis result depends on a long learning curve; however, for trained physicians, it is a sensitive and specific tool for the early detection of melanomas and other skin tumors. The main current indications for this type of examination are isolated assessment of dubious melanocytic lesions, investigation of pigmented or non-pigmented lesions suspected of skin cancer, and preoperative delimitation of excision margins or post-treatment follow-up.^1^, ^2^

The main correlations between histopathological findings and RCM were first described in the 1990s.^3^, ^4^ Since then, hundreds of articles have been published, showing the importance of RCM as a complementary tool in the diagnosis of melanoma and non-melanoma skin cancers. RCM is also used in the evaluation of normal skin, melanocytic nevi, and benign non-melanocytic lesions. The definition and terms related to RCM criteria were established by a consensus published in the medical literature in 2007 and updated in 2009.^5^, ^6^ The standardization of RCM terminology for both melanocytic and non-melanocytic lesions was recently published in the English language, after a systematic review of the terms used to describe the main findings in RCM in original articles during the years 1995‒2017.^7^, ^8^

Despite the numerous publications on RCM in English and the growing number of Brazilian dermatologists using this new tool, to date, there is no official terminology in Portuguese. The objective of the present study is to propose a terminology in Brazilian Portuguese for the description of the RCM exam, generating the first Brazilian consensus on terms related to normal skin and cutaneous tumors, which will allow Brazilian dermatologists to communicate more accurately and homogeneously, facilitating the exchange of information and knowledge in the area.

## Methods

Ten specialists (nine dermatologists and one dermatopathologist) were selected according to their publications, participation in studies, and years of RCM technique use in the diagnosis of skin tumors. The selection included professionals from different institutions and different states of Brazil, determining heterogeneity of the group, a crucial characteristic in this type of study.

Participants were invited as panelists via electronic communication and their participation in this project was completely voluntary and had no financial support.

The modified Delphi method was used for the creation of the consensus,^9^ in 3 steps. As a support tool for data collection, electronic forms were generated using the Google Forms tool (normal skin, melanocytic lesions, and non-melanocytic lesions). The forms were sent to each participant by e-mail and the responses were received anonymously.

In the first step, a list of terms used in RCM for normal skin and melanocytic and non-melanocytic tumors was created, encompassing benign and malignant lesions, based on related terms in the English language used in the 2007 consensus, revised in 2021, and in systematic reviews published in 2019 and 2020.^5^, ^7^, ^8^, ^10^ Each participant had to choose the best corresponding term in the Portuguese language, commonly used for describing the structure in question or suggest another appropriate term, in addition to those that were discriminated in each list.

After analyzing the answers and results of the first step, forms were created listing most voted or suggested terms for each structure, only for the terms with agreement inferior to 70%.

In the third step of the process, two meetings were held with the virtual presence of the participants to analyze the terms which hadn’t reached 70% in the second step, with the purpose of obtaining a definitive consensus on the term in question. The first meeting was held with the presence of 100% of the specialists and the results of the terms for normal skin and melanocytic lesions were analyzed. The second meeting was held with the presence of 80% of the specialists and the terms for non-melanocytic lesions were analyzed.

## Results

**Figure 1:**
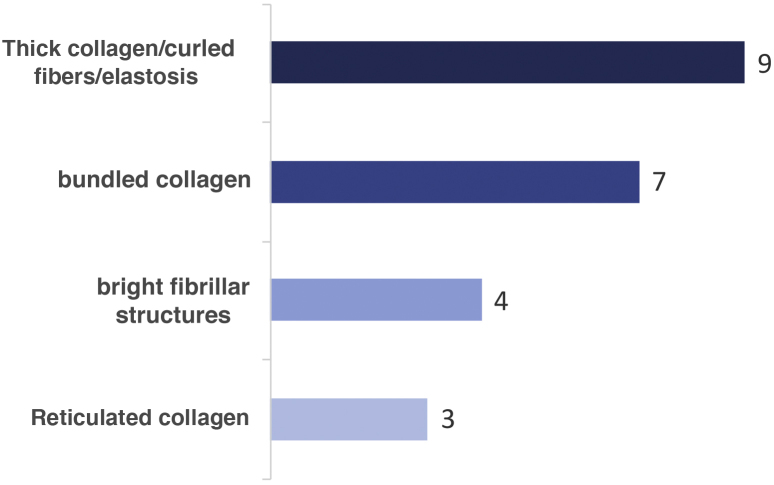
Terms used in RCM to describe different aspects of collagen in the papillary dermis and number of participants using each term

**Figure 2:**
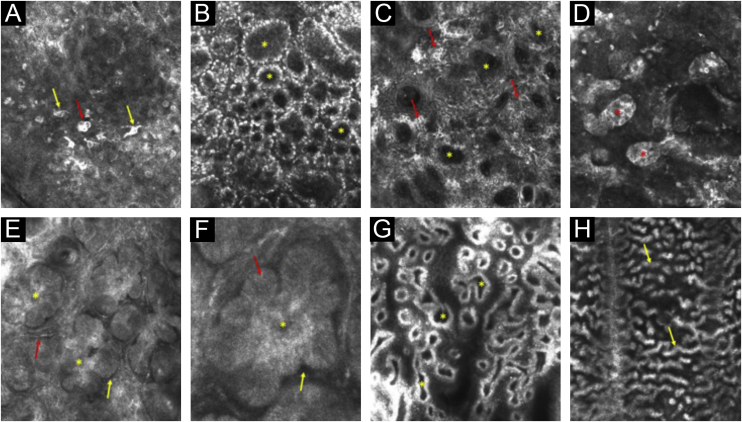
RCM images illustrating some key consensus terms. (A) Polymorphic pagetoid cells – round (red arrow) and dendritic (yellow arrows). (B) Ringed pattern – edged papillae (asterisks). (C) Meshwork pattern – non-edged papillae (asterisks) and dendritic cells in the interpapillary spaces (red arrows). (D) Dense nests (asterisks). (E) Tumor islands (asterisks), peritumoral dark cleft (yellow arrow) and dilated linear blood vessels (red arrow). (F) Tumor island (asterisk), peripheral palisading (red arrow) and peritumoral dark cleft (yellow arrow). (G) Polycyclic and polymorphic papillae (asterisks). (H) Cords and bulbous projections (yellow arrows)

As a result of the consensus, the most voted terms which were considered the most suitable for use in the Portuguese language will be presented here. The terms are listed in Table 1, Table 2, Table 3, Table 4, Table 5, along with the corresponding description of the confocal microscopy findings and correlation with histology, for normal skin, melanocytic and non-melanocytic lesions.^11^ Fig. 1 shows the terms used to describe the different aspects of collagen in the papillary dermis and the number of participants using each of the mentioned terms. Fig. 2 illustrates the main terms considered for the description of melanocytic and non-melanocytic lesions.

**Table 1:** Result of the expert consensus – RCM terminology for describing normal skin

Term	Agreement	Definition in RCM
Stratum corneum	80%	Most superficial layer of the epidermis. It is brighter than the other layers of the epidermis, basically composed of keratin. Corneocytes measuring 10 to 30 micrometers, flattened, polygonal, anucleated and with a dark outline.
Stratum granulosum	80%	Located 15 to 20 micrometers from the surface and composed of keratinocytes, measuring 25 to 35 micrometers, polygonal, with a round dark central nucleus and surrounded by granular bright cytoplasm (organelles and keratohyalin granules).
Stratum spinosum	80%	Located 20 to 100 micrometers from the surface with higher density of keratinocytes, which are 15 to 25 micrometers in size, polygonal, with a dark rounded central nucleus and bright cytoplasm.
Basal layer	100%	Located 50 to 100 micrometers from the stratum corneum. Single layer with grouping of rounded refractive cells, above the papillary dermis. Cell nuclei are bright due to the presence of a melanin layer over them. Isolated melanocytes can be seen in the basal layer - rounded, fusiform and more rarely with dendritic projections.
Dermoepidermal junction (DEJ)	100%	Presence of dark rounded areas (top of dermal papillae) surrounded by bright keratinocytes. Some blood vessels can be seen in the dermal papillae with their real-time flow.
Superficial dermis	100%*	Located 100 to 150 micrometers from the surface. It shows collagen fibers forming a reticular pattern. Deeper, thicker parallel bundles of collagen are seen.
Skinfolds (Dermatoglyphs)	100%	Non-refractive (dark) grooves that separate “islands” of keratinocytes in the stratum corneum
Honeycomb pattern	100%	Normal pattern of the epidermis in the stratum granulosum and stratum spinosum ‒ polygonal keratinocytes with well-demarcated bright outlines, uniform in outline shape, size and thickness
Cobblestone Pattern	100%	Normal pattern of the epidermis in the stratum basale ‒ well-defined bright rounded cells separated by a less refractive polygonal outline.
Edged papillae	70%	Normal dermal-epidermal junction pattern ‒ less refractive (dark) papillary dermis surrounded by ring of bright cells
Hair follicle	100%	Structure characterized by cells of different sizes according to their differentiation ‒ small in the stratum basale and large in the central strata, with a round or polygonal shape.
Hair shaft	100%*	Elongated tubular or cylindrical structure without cellularity with high refractivity and uniformly bright.
Blood vessels	100%	Structures seen in dermal papillae, darker than the surrounding stroma, where the movement of rounded, bright cells can be seen in real time. They may have a canalicular appearance ‒ straight or tortuous or a rounded appearance. They can also be described according to their density and diameter.
Collagen	100%*	Bright fibrillar elongated structures without cellularity and without movement, distributed side by side in the dermis. They are randomly distributed in the papillary dermis and in parallel bundles in the reticular dermis.

* After the third step of the analysis, in a meeting with the virtual presence of all participants. Note: The table in Portuguese is available on the journal website (https://www.anaisdedermatologia.org.br/)

**Table 2:** Result of the expert consensus – RCM terminology for describing melanocytic lesions

Term	Agreement	Definition in RCM	Histopathological correlation
**Pagetoid cells**	100%	Presence of large, nucleated cells in the epidermis, twice the size of keratinocytes, with dark nuclei and bright cytoplasm (shape not specified)	Melanocytes in the suprabasal layers of the epidermis
**Polymorphic Pagetoid Cells**	90%	Presence of cells in the epidermis that are round and dendritic (variability of the aspect of pagetoid cells)	Melanocytes in the suprabasal layers of the epidermis
**Round pagetoid cells**	70%	Presence of bright nucleated cells with dendritic processes in the epidermis (variability of the aspect of pagetoid cells)	Melanocytes in the suprabasal layers of the epidermis
**Dendritic Cells**	100%*	Presence of nucleated and round cells with dark nucleus and bright cytoplasm in the epidermis (variability of the aspect of pagetoid cells)	Melanocytes in the suprabasal layers of the epidermis
**Dark Pagetoid Cells**	70%	Presence of low refractivity cells characterized as dark structures similar to “holes” in the epidermis (variability of the aspect of pagetoid cells)	Melanocytes in the suprabasal layers of the epidermis
**Atypical Cells**	80%	Presence of atypical/irregular cells in the epidermis without specifying the format	Melanocytes in the suprabasal layers of the epidermis
**Dendritic Structures**	80%	Presence of dendrites in the epidermis – numerous interspersed bright lines originating from dendritic cells, with the cell body not always visible	Dendritic projections of melanocytes or Langerhans cells in the epidermis
**Atypical cells infiltrating follicular structures**	80%	Infiltrating dendritic/round cells in the inner part of the hair follicle	Infiltration of the hair follicle and adnexal structures by atypical melanocytes - aspect observed in melanomas of the lentigo maligna type
**Atypical Honeycomb Pattern/Architectural disorder of the epidermis**	50%/50%	Atypical keratinocytes or keratinocyte disarray	Keratinocytes with variation in size and shape
	100%* for the use of both terms		
**Epidermal Granularity**	90%	Presence of bright granular particles in the epidermis, similar to “dust” (“speckled” appearance)	Extracellular melanin granules in the epidermis
**Non-edged papillae**	90%	Irregular outline of dermal papillae with no rim of bright cells without bright cell edges	Enlarged interpapillary spaces with the presence of large atypical melanocytes
**Non-visible dermal papillae**	100%*	Loss of papillary contour / papillary architecture or non visible dermal-epidermal transition	Disordered proliferation of melanocytes determining alteration of the epidermal ridges (flattened epidermis)
**Large dendritic or round nucleated cells, in the DEJ**	100%*	Presence of atypical cells in the DEJ - large, bright, round or dendritic (>50 micrometers) nucleated cells with unusual outline (triangular/star-shaped) or eccentric large nucleus	Proliferation of atypical melanocytes as solitary units in the DEJ
**Spindle Cells**	90%	Atypical cells with different shapes in the DEJ	Proliferation of atypical melanocytes in the DEJ
**Focal increase of atypical melanocytes and nests**	70%	Presence of atypical cells in the DEJ with additional characteristics such as dense nests, sparse nests, bright cells of varying sizes, etc.	Proliferation of atypical melanocytes or nests of atypical melanocytes in the DEJ
**Junctional nests**	90%	Presence of regular round junctional cellular aggregates	Typical melanocyte nests in the DEJ
**Junctional Thickening**	80%	Presence of tubular/ elongated junctional aggregates	Elongated junctional melanocyte nests
**“mitochondria-like” structures**	100%	Dendritic cells that project from the epidermis towards the dermis forming “bridges”	Structures observed in horizontal histopathological sections: increased atypical melanocytes around the dermal papillae, projecting towards the center of the dermal papillae forming “bridges”^11^
**Short Interconnections**	100%	Junctional thickening and nests around the papillae	Elongated nests of junctional melanocytes that can form “bridges” between interpapillary ridges
**Presence of a large amount of bright cells in the DEJ (dendritic and/or round nucleated cells): sheet of cells**	80%	Proliferation of cells in the DEJ, distributed on the same plane, in a non-aggregated form, and with blurring of dermal papillae	Lentiginous proliferation of atypical melanocytes in the DEJ, mainly in melanomas in areas with sun damage
**Medusa head–like structures**	90%	Elongated structures that protrude from hair follicles - distributed around the entire follicle perimeter	
**Nucleated round cells in the dermis**	100%*	Presence of solitary melanocytes in the papillary dermis – rounded/oval with well-defined bright cytoplasm and central dark nucleus	Presence of atypical melanocytes in the papillary dermis
**Dense nests**	70%	Presence of cohesive nests of melanocytes in the papillary dermis – well-defined, compact aggregates of monomorphic cells with easily identified individual edges	Nests of round/oval junctional or dermal melanocytes
**Dense and sparse nests**	70%	Presence of irregular/disconnected nests of melanocytes in the papillary dermis	Nests of atypical melanocytes
**Cerebriform nest**	80%	Presence of “cerebriform” aggregates in the dermis	Nodular aggregates of atypical melanocytes in melanomas with a dermal component
**Melanophages**	100%*	Irregularly-shaped bright cells with poorly-defined edges and usually no visible nucleus	Melanophages in the papillary dermis
**Bright dots / Bright particles**	50%/50%	Small bright particles in the dermis	Inflammatory cells in the dermis, other than melanophages
	100%* for the use of both terms		
**Irregular vessels/ Numerous vessels of increased caliber**	50%/50%	Presence of prominent vessels in the papillary dermis	Dilated and increased vascularity in the superficial dermis
	100%* for the use of both terms		
**Ringed pattern**	70%	Papillae with edges well demarcated by the presence of bright cells, forming “rings”	Presence of junctional melanocytes arranged side by side or in small nests
**Meshwork pattern**	100%	Enlarged interpapillary spaces, predominantly consisting of junctional thickening and/or non-edged papillae	Irregular proliferation of junctional nests of melanocytes, forming bridges between epidermal ridges
**Clod pattern**	100%	Predominance of dense and compact melanocyte nests or aggregates in the superficial dermis	Proliferation of melanocyte nests in the dermis
**Mixed pattern**	100%	Combination of 2 or more patterns seen in melanocytic neoplasms with a junctional and a dermal component	
**Non specific pattern**	80%	Loss of recognizable pattern in the DEJ, usually associated with melanocytic proliferations with abrupt or imprecise epidermal/dermal transition	
**Asymmetry**	70%	The distribution of structures seen on confocal microscopy is different in the two halves of the lesion	Does not apply
**Nests at the periphery of the lesion**	90%	Presence of junctional or dermal nests distributed around the periphery of the lesion	
**Sharp border cutoff**	70%	Precise demarcation between the edge of the lesion and the skin at the periphery, a pattern often seen in Spitz nevi	Does not apply

* After the third step of analysis, in a meeting with the virtual presence of all participants. Note: The table in Portuguese is available on the journal website (https://www.anaisdedermatologia.org.br/)

**Table 3:** Result of the expert consensus – RCM terminology for the description of basal cell carcinomas of the skin

Term	Agreement	Definition in RCM	Histopathological Correlation
Atypical Honeycomb Pattern	90%	Keratinocytes with varying size nuclei, pleomorphism, architectural disarray, and parakeratotic nuclei	Epidermal keratinocytes atypia
Polarization of epidermal nuclei	70%	Elongated, monomorphic, basaloid nuclei, aligned on the same axis	Frontal view of the upper part of the palisaded tumor cord or nest
Epidermal shadow	100%	Large, structureless area, poorly-defined edges, interrupting the normal epidermis and corresponding to the horizontal cleft	Optical effect of the frontal view of an underlying tumor nest
Ulceration	100%	Disruption of the bright skin surface and underlying layers of the epidermis, seen as a dark area with or without shiny amorphous or fibrillar debris	Ulceration
Prominent nucleoli	100%	Visible nucleolus in the nucleus of elongated cells of basal cell carcinoma	Prominent nucleolus in the keratinocyte nuclei
Onion-like structures	70%	Dark rounded spaces, centered by bright refractive material	Epidermal cysts (milia)
Tumor island	100%*	Packed cells corresponding to tumor islands with high refractivity	Nests of basaloid cells in the DEJ or superficial dermis, a characteristic usually associated with the nodular type
Peripheral palisading	80%	Cells with elongated and palisaded nuclei at the periphery of the tumor parenchyma	Basaloid cells organized at the periphery of tumor nests
Dark peritumoral cleft	70%	Dark spaces (low refractivity) around tumor nests	Probable presence of mucin between the tumor parenchyma and the surrounding stroma (clear spaces)
Cord-like structures	100%*	Compacted tumor cells forming trabeculae	Nests of basaloid cells connected to the DEJ, a characteristic highly associated with the superficial type
Dendritic cells or dendrites	100%*	Bright, fine or coarse dendritic structures within the tumor islands, often associated with clearly visible nucleated cells	When inside the tumor, they most frequently correspond to melanocytes and, less frequently, to Langerhans cells
Dark silhouettes	100%	Tumor island or area of low refractivity delineated by the bright collagen bundles of the surrounding dermis (basaloid islands)	Nests of basaloid cells in the superficial or deep dermis (not visible due to loss of resolution) – associated with the infiltrative type
Solar Elastosis	100%	Bright, irregular bundles and “lace-like” structures	Degeneration of elastic fibers in skin chronically exposed to the sun
Melanophages	70%	Irregularly shaped bright cells, with poorly-defined edges and usually no visible nucleus, distributed inside and outside tumor islands	Melanophages in the superficial dermis
Thickened collagen bundles	90%	Increased number of fibrous bundles arranged in parallel around the tumor	Collagen response to the tumor in the surrounding stroma
Linear or convoluted dilated blood vessels	80%	Increased number of dilated blood vessels with occasional rolling of leukocytes. Vessels often horizontal (parallel) to the surface	Dilated blood vessels parallel to the epidermal surface
Bright spots or Bright particles	100%*	Small shiny particles in the dermis	Inflammatory cells in the dermis, other than melanophages

* After the third step of analysis, in a meeting with the virtual presence of all participants. Note: The table in Portuguese is available on the journal website (https://www.anaisdedermatologia.org.br/)

**Table 4:** Result of the expert consensus – RCM terminology for describing Squamous Cell Carcinomas (SCC) of the skin and actinic keratoses

Term	A	Definition in RCM	Histopathological Correlation
**Hyperkeratosis**	100%	Thickening of the stratum corneum (> 15 micrometers)	Hyperkeratosis
**Parakeratosis**	100%	Nucleated cells appearing as central dark nuclei with a bright outline, corresponding to corneocytes	Parakeratosis
**Polygonal nucleated cells at the stratum corneum**	100%*	Polygonal, highly refractive white structure, measuring 30 to 40 micrometers in diameter in the stratum corneum	“Detached” corneocytes
**Orthokeratosis**	100%	Hyperkeratosis without parakeratosis	Orthokeratosis
**Scale**	100%	Variably refractile, amorphous material in stratum corneum	Hyperkeratosis
**Atypical Honeycomb Pattern**	70%	Keratinocytes with varying size nuclei, pleomorphism, architectural disarray, and parakeratotic nuclei	Atypia of epidermal keratinocytes
**Architectural Disarray**	70%	Highly disorganized epidermal pattern in which the honeycomb architecture is no longer identifiable	Marked variation in cell and nuclear size and shape of keratinocytes
**Keratinocyte pleomorphism**	70%	Variation in cell and nuclear size and shape, an aspect mainly related to individual cell morphology and not to the epidermal pattern	Keratinocyte pleomorphism
**Targetoid cell or dyskeratotic cell**	100%*	Large cells with bright center and peripheral dark halo or large cells with dark center and bright rim surrounded by dark halo	Dyskeratotic keratinocytes
**Multinucleated keratinocytes**	80%	Large cells with clusters of bright nuclei	Multinucleated keratinocytes
**Spongiosis**	100%	Enlargement of bright intercellular spaces due to accumulation of fluid between keratinocytes	Spongiosis
**Exocytosis**	100%	Inflammatory cells presenting as highly refractive structures in the epidermis	Exocytosis
**Dendritic cells in the epidermis**	90%	Bright cells with elongated, branching structures projecting from the fusiform cell body, seen in the stratum granulosum and stratum spinosum of the epidermis	Langerhans cells infiltrating the epidermis, seen in pigmented SCC or pigmented actinic keratosis
**Ulceration**	70%	Dark areas with irregular outlines, filled with amorphous material, cell debris and small particles	Ulceration
**Corneal Pseudocysts**	90%	Highly refractive, round, large, and circumscribed intraepidermal structures	Intraepidermal cysts
**Edged Papillae**	70%	Multiple scattered dermal papillae demarcated by a rim of bright cells - in pigmented SCC, the edged papillae are mostly peripheral in location with widened interpapillary spaces	Atypical, pigmented keratinocytes
**Keratin pearl**	100%	Whorl-shaped accumulation of keratin appearing as a highly refractive, speckled structure in the dermis	Keratinization
**Convoluted glomerular vessels**	100%	Coiled canalicular vessels	Abnormal vessels
**Linear vessels**	100%	Vessels oriented parallel to the imaging plane	
**“S”-shaped vessels**	90%	Round-to-oval vessels with increased tortuosity at the center of the dermal papillae, S-shaped at the lower papillary dermis	
**Increased blood vessels dilatation**	100%	Elongated and dilated vessels in the dermis	
**Dilated looping blood vessels within papillae**	70%	Dilated blood vessels inside the dermal papilla, perpendicular to the horizontal plane of the image with a “buttonhole” appearance	Abnormal neoformed vessels
**Increased number of blood vessels**	100%	Presence of more than 5 blood vessels per 0.5 × 0.5 mm	Neovascularization
**Solar elastosis**	90%	Thick, highly refractive collagen bundles interspersed with moderately refractive, lace-like elastic fibers	Degeneration of elastic fibers in skin chronically exposed to the sun
**Tumor nests in the dermis**	100%	Intradermal tumor cell nests with adjacent collagen thickening – seen in invasive SCC	Tumor nests in the dermis
**Melanophages**	70%	Glossy cells in the dermis, irregularly shaped with poorly-defined borders and usually no visible nucleus	Melanophages in the dermis
**Round/polygonal nucleated cells in the dermis**	100%	Round and/or polygonal cells with bright edges and central dark nucleus, located in the superficial dermis, described in superficially invasive SCC	Atypical keratinocytes in the superficial dermis
**Inflammatory cells**	70%	Highly refractive structures, measuring 8 to 10 micrometers in diameter, located in the epidermis or dermis	Lymphocytes and neutrophils

* After the third step of the analysis, in a meeting with the virtual presence of all participants. Note: The table in Portuguese is available on the journal website (https://www.anaisdedermatologia.org.br/)

**Table 5:** Result of the consensus among specialists –RCM terminology for describing seborrheic keratoses, solar lentigines and lichenoid keratoses

Term	Agreement	Definition in RCM	Histopathological Correlation
**Corneal pseudocyst**	80%	Homogeneous bright, non edged, well demarcated intraepidermal areas, surrounded by a dark halo	Intraepidermal keratin cysts
**Keratin-filled invaginations or sulci and gyri on the surface**	100%*	Rounded and longitudinal invaginations on the surface of the lesion, filled with amorphous material of varying brightness	Papillomatous epidermis filled with keratin
**Cerebriform aspect**	90%	Round, linear structures, darker than the surrounding epidermis, resembling the brain surface (sulci and gyri)	Papillomatous epidermis
**Typical honeycomb pattern**	80%	Regular honeycomb pattern – cells and nuclei have regular size and shape	Regular epidermis
**Epidermal projections**	100%	Projections from the epidermal surface of the lesion	Projections of the epidermal ridges
**Homogeneous small, bright cells**	90%	Small, homogeneous, bright cells in the stratum basale	Pigmented keratinocytes in the stratum basale
**Polycyclic and polymorphic papillae**	100%	Compacted, round, polymorphic dermal papillae, often with pigmented keratinocytes, seen in the DEJ	Connected, elongated interpapillary ridges with pigmented keratinocytes
**Cords and bulbous projections**	80%	Bright, elongated tubular structures (cords), with bulbous projections, in the DEJ	
**Mixed vascular pattern**	100%	Prominent vascular pattern with dilated round and linear vessels, perpendicular and parallel to the surface, respectively	Neovascularization
**Melanophages**	70%	Irregularly shaped bright cells with poorly-defined edges and usually no visible nucleus	Melanophages in the dermis
**Small, bright particles**	70%	Small, round, bright structures with no visible nucleus	Presence of inflammatory cells (lymphocytes and neutrophils)

DEJ, Dermal-epidermal junction.

* After the third step of the analysis, in a meeting with the virtual presence of 80% of the participants. Note: The table in Portuguese is available on the journal website (https://www.anaisdedermatologia.org.br/)

## Discussion

RCM aims to provide instantaneous, real-time, high-resolution *in vivo* images, allowing the observation of microanatomical structures (cells, nuclei and tissue architecture) of the skin at a resolution close to the one of histopathology. This tool has been widely used in the diagnosis of melanoma and non-melanoma skin tumors. The images obtained by this method are from planes parallel to the skin, similar to dermoscopy and different from conventional histological sections.^3^, ^4^

*In vivo* confocal microscopy has some differences when compared to conventional histopathology. The examination is painless and non-invasive, with no tissue damage. RCM provides black-and-white images as opposed to the pink and purple staining seen in hematoxylin-eosin stained histological slides. The skin is not altered by the fixation or staining process, minimizing artifacts or disruption of the original tissue structure. Currently, confocal microscopes provide a unique opportunity for non-invasive examination of the skin without the need for fluorescent markers or tissue dyes. The contrast in confocal images occurs due to natural variations in the refractive index of organelles, and microstructures in different skin layers.^2^, ^3^, ^4^

RCM may eventually help to prevent the unnecessary excision of benign lesions, guide biopsy of suspicious pigmented lesions, map pre- and intraoperative tumor margins, and monitor therapeutic response.^12^ The interpretation of RCM images is a difficult task with a long learning curve. Moreover, the inconsistent use of terms in scientific meetings and the lack of terminology standardization in Brazilian Portuguese probably represent an even greater challenge for beginners in the incorporation and training in this technology for beginners.

Terminology standardization, with the use of non-redundant terms, facilitates uniformity in the preparation of the RCM exam report and communication between experienced professionals in the area. Aiming at improving the consistency of the use of RCM terms through standardized language, the authors carried out a terminology consensus in Brazilian Portuguese with the creation of a concise and unified glossary for normal skin, melanocytic lesions, and non-melanocytic lesions.

This consensus was used as the main research basis for two studies recently published by Navarrete-Dechent et al. at the American Academy of Dermatology, where they performed a systematic review of RCM terminology used in the literature for both melanocytic and non-melanocytic lesions. The authors gathered all RCM terms described in original articles, and identified probable synonyms with similar definitions and histopathological correlation, which were grouped together.^7^, ^8^ Therefore, redundant terms were unified and the list of terms was reduced by approximately 50%, facilitating the creation of a concise glossary and, consequently, improve learning and clinical application of RCM by dermatologists.

RCM is a technology that has gained prominence in Brazil since 2009, mainly in the cutaneous oncology scenario, when the first exams were performed. From then on, the nomenclature used is the one described in the English language, generating great difficulty both in learning and in the preparation and interpretation of reports.

Brazilian professionals from different states, active and experienced in the field of RCM, gathered to standardize the most frequently used descriptive terms for the evaluation of normal skin, melanocytic lesions, and non-melanocytic lesions. It is believed that the creation of this glossary in Brazilian Portuguese will be very useful as a guide for the description of RCM images and also as a didactic tool for beginners using this technology.

The methodology used in this study included the possibility of evaluating the nomenclature in three steps when agreement did not reach at least 70% of the participants. The terms that required two or more steps to reach the desired consensus were related to questions of semantics, subjectivity regarding their description and/or variations between descriptive and metaphorical terms. The consensus was reached with emphasis on its practical use and how easy it was to understand the meaning of the term in relation to its histopathological correspondence.

This Brazilian consensus represents an opportunity for dermatologists and physicians specializing in cutaneous oncology to become familiar with RCM, in addition to the possibility of disseminating the technique in clinical and research settings.

## Conclusion

The authors expect that this consensus can be applied by Brazilian professionals to expand the learning and use of RCM in different scenarios in the medical field, facilitating debates in symposiums, congresses, and scientific meetings and contributing to national and international publications.

## Financial support

None declared.

## Authors' contributions

Juliana Casagrande Tavoloni Braga: Design and planning of the study; drafting and editing of the manuscript; collection, analysis, and interpretation of data; critical review of the literature; approval of the final version of the manuscript.

Carlos B. Barcaui: Effective participation in research orientation; collection of data; critical review of important intellectual content; approval of the final version of the manuscript.

Ana Maria Pinheiro: Collection of data; critical review of important intellectual content; approval of the final version of the manuscript.

Ana Maria Fagundes Sortino: Collection of data; critical review of important intellectual content; approval of the final version of the manuscript.

Cristina Martinez Zugaib Abdalla: Collection of data; critical review of important intellectual content; approval of the final version of the manuscript.

Gabriella Campos-do-Carmo: Collection of data; critical review of important intellectual content; approval of the final version of the manuscript.

Gisele Gargantini Rezze: Collection of data; critical review of important intellectual content; approval of the final version of the manuscript.

Juan Piñeiro-Maceira: Collection of data; critical review of important intellectual content; approval of the final version of the manuscript.

Lilian Licarião Rocha: Collection of data; critical review of important intellectual content; approval of the final version of the manuscript.

Marcus Maia: Collection of data; critical review of important intellectual content; approval of the final version of the manuscript.

Bianca Costa Soares de Sá: Design and planning of the study; data survey; analysis and interpretation of data; drafting and editing of the manuscript; critical review of the literature; approval of the final version of the manuscript.

## Conflicts of interest

None declared.
